# Hybridization and low genetic diversity in the endangered Alabama red‐bellied turtle (*Pseudemys alabamensis*)

**DOI:** 10.1002/ece3.8964

**Published:** 2022-06-03

**Authors:** Nickolas Moreno, Andrew Heaton, Kaylin Bruening, Emma Milligan, David Nelson, Scott Glaberman, Ylenia Chiari

**Affiliations:** ^1^ 5557 Department of Biology University of South Alabama Mobile Alabama USA; ^2^ 3298 Department of Biology George Mason University Fairfax Virginia USA; ^3^ Grand Bay National Estuarine Research Reserve Mississippi Department of Marine Resources Moss Point Mississippi USA; ^4^ 3298 Department of Environmental Science and Policy George Mason University Fairfax Virginia USA

**Keywords:** conservation, endemism, Hubbs principle, microsatellites, mitochondrial DNA, southeastern United States, turtles

## Abstract

*Pseudemys alabamensis* is one of the most endangered freshwater turtle species in the United States due to its restricted geographic distribution in coastal Alabama and Mississippi. Populations of *P. alabamensis* are geographically isolated from one another by land and saltwater, which could act as barriers to gene flow. It is currently unknown how differentiated these populations are from one another and whether they have experienced reductions in population size. Previous work found morphological differences between Alabama and Mississippi populations, suggesting that they may be evolutionarily distinct. Other *Pseudemys* turtles such as *P. concinna* and *P. floridana* occur naturally within the same geographic area as *P. alabamensis* and are known to hybridize with each other. These more abundant species could threaten the unique genetic identity of *P. alabamensis* through introgression. In order to evaluate the endangered status of *P. alabamensis* and the level of hybridization with other species, we used mitochondrial and nuclear microsatellite markers to assess genetic variation within and among populations of *P. alabamensis* throughout its range and estimate admixture with co‐occurring *Pseudemys* species. In *P. alabamensis*, we found no variation in mitochondrial DNA and an excess of homozygosity in microsatellite data. Our results show genetic differentiation between Alabama and Mississippi populations of *P. alabamensis*, and low estimated breeding sizes and signs of inbreeding for two populations (Fowl River, Alabama and Biloxi, Mississippi). We also found evidence of admixture between *P. alabamensis* and *P. concinna*/*P. floridana*. Based on our results, *P. alabamensis* is highly endangered throughout its range and threatened by both low population sizes and hybridization. In order to improve the species’ chances of survival, focus should be placed on habitat preservation, maintenance of genetic diversity within both the Mississippi and Alabama populations, and routine population‐monitoring activities such as nest surveillance and estimates of recruitment.

## Graphical abstract

*Pseudemys alabamensis* is one of the most endangered freshwater turtle species in the United States. Populations of *P. alabamensis* are geographically isolated from one another by land and saltwater, which could act as barriers to gene flow, resulting in the evolution of unique populations. Other *Pseudemys* turtles such as *P. concinna* and *P. floridana* occur naturally within the same geographic area as *P. alabamensis* and are known to hybridize with each other. These more abundant species could threaten the unique genetic identity of *P. alabamensis* through introgression. We used mitochondrial and nuclear microsatellite markers to assess genetic variation within and among populations of *P. alabamensis* throughout its range and estimate admixture with co‐occurring *Pseudemys* species. We found that *P. alabamensis* is highly endangered throughout its range and threatened by both low population sizes and hybridization. Conservation measures must be developed to prevent further reduction and extinction of this endangered species.

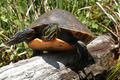

## 1 INTRODUCTION

**FIGURE 1:**
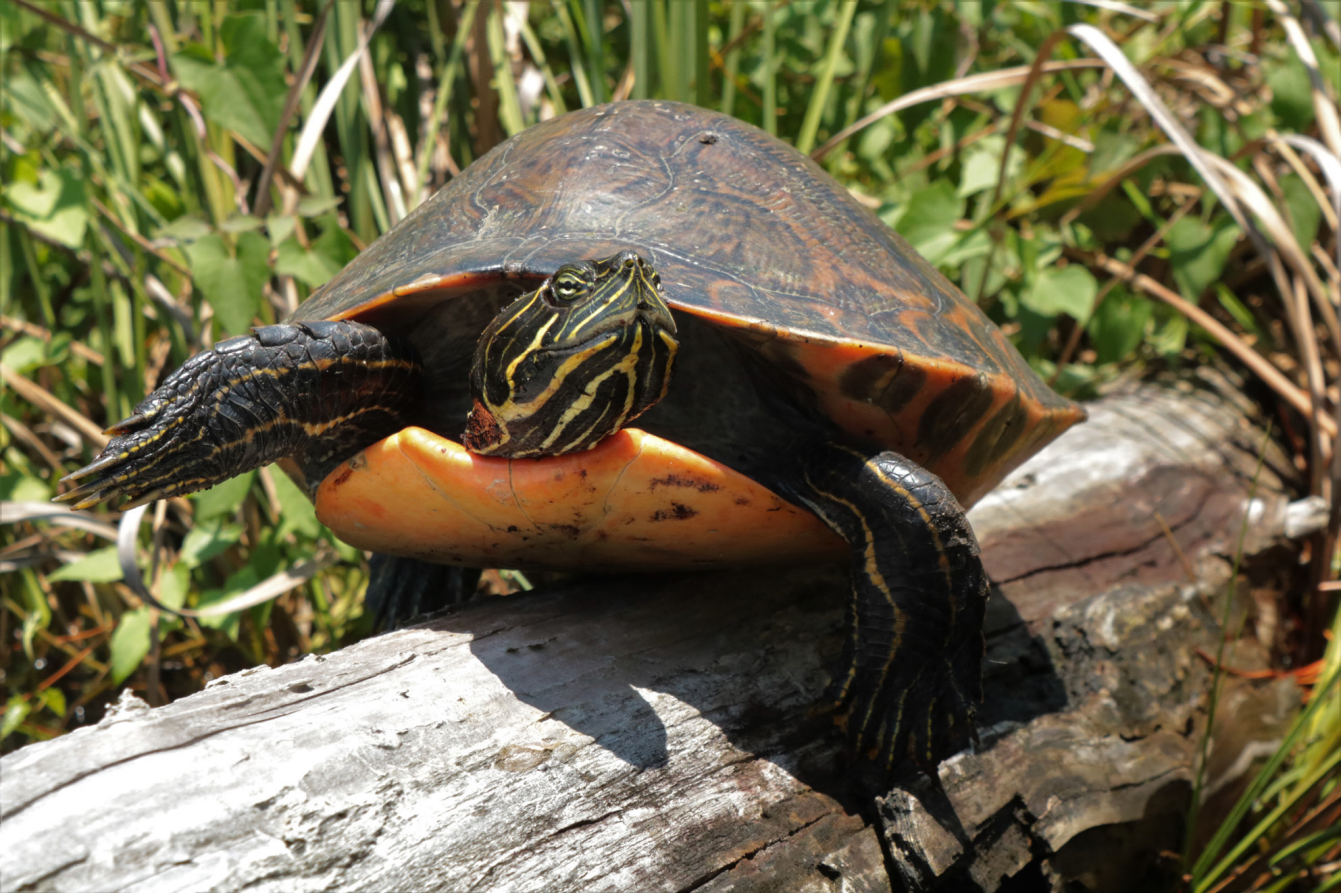
*Pseudemys alabamensis* in its natural environment. Photo credit: Nickolas Moreno

The southeastern United States is a biodiversity hot‐spot, harboring higher levels of endemic species than other areas of the country (Jenkins et al., 2015). Alabama, in particular, has a high concentration of regionally endemic species, especially freshwater turtles, and occurs within one of three global turtle priority areas for conservation (Buhlmann et al., 2009; Lydeard & Mayden, 1995). Freshwater turtles are a conservation concern worldwide, with >60% of species classified as threatened (Buhlmann et al., 2009). While some turtle species in the southeastern US are not currently imperiled, others have multiple risk factors for extinction such as low population size and restricted habitat range (IUCN, 2001; Mace et al., 2008; Purvis et al., 2000). The Alabama red‐bellied turtle (*Pseudemys alabamensis*) is among the most at‐risk turtle species in the US and is considered by some to be “the most endangered turtle on the continent” (Spinks et al., 2013). Although it is classified as endangered by both the US Fish and Wildlife Service (USFW, 1987) and the International Union for Conservation of Nature (IUCN) Red List, studies on this species across its entire distribution are lacking. This dearth of information prevents development of targeted management and conservation actions. Although *P*. *alabamensis* does occur within some protected areas (Heaton et al., 2021), there are currently no specific survey activities or targeted management actions to ensure monitoring and protection of this species (Figure 1).

**FIGURE 2:**
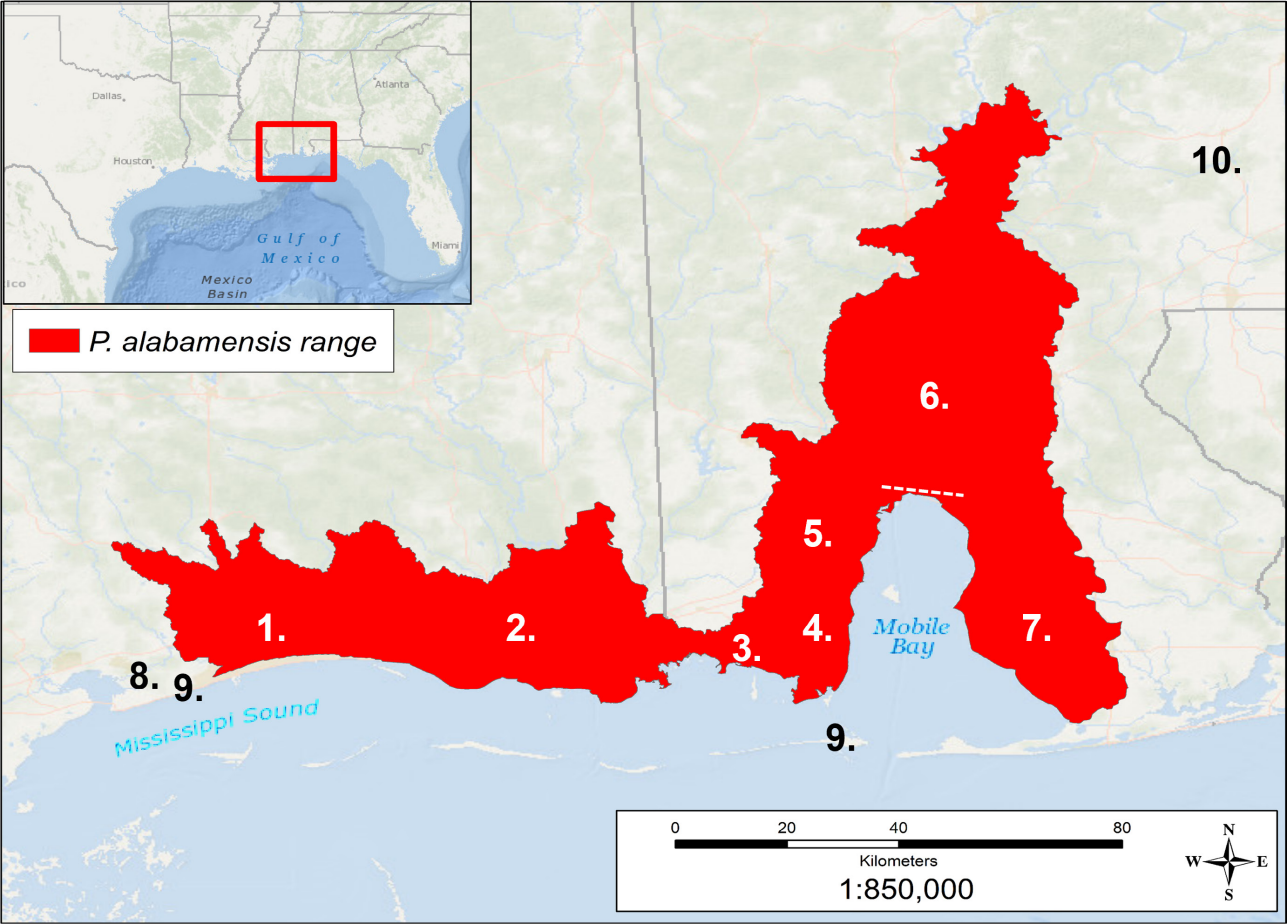
Projected range of *P*. *alabamensis* based on GIS‐defined hydrologic unit compartments (HUCs) created around capture locations from this study along with data from Nelson (1994, 1995, 1996, 1997, 1998), Leary et al. (2003), and Jackson et al. (2012). Approximate locations of rivers sampled within the range are marked by numbers as follows (numbers as in Table 1): 1. Biloxi, 2. Pascagoula, 3. Bayou La Batre, 4. Fowl River, 5. Dog River, 6. Mobile‐Tensaw Delta (Mobile Bay Causeway [US HWY 98] indicated with dashed line), 7. Weeks Bay, 8. Wolf River (single individual), 9. Waif individuals, 10. Location of extirpated population near Little River State Park

*Pseudemys alabamensis* is threatened by habitat modification, including dredging, road‐kill of adults and juveniles, and competition with other species (Nelson et al., 2009). Turtles may also be used for shooting practice (Alexander, 2018). This species has a very limited distribution and is found exclusively in coastal rivers along Mobile Bay in Alabama and the Mississippi Sound (Figure 2) (Leary et al., 2008). An isolated population once existed further inland in southwestern Alabama not far from Little River State Park, but has since been extirpated (Mount, 1975). The freshwater bodies currently inhabited by *P*. *alabamensis* are separated by land and saltwater, which likely prevents substantial movement of individuals between river populations. In fact, although *P*. *alabamensis* shows some tolerance to brackish water, it does not occur in saltwater and is known to only disperse on land for nesting purposes at distances between 30 and 130 m from water bodies (Nelson et al., 2009). Some morphological differences have been previously noted between Alabama and Mississippi populations of *P*. *alabamensis,* such as the dorsal width of the cervical scute (Leary et al., 2003), supporting the existence of isolated populations within this species.

Despite the small range and fragmented populations of *P*. *alabamensis*, virtually nothing is known about key factors needed for developing a species survival plan such as population size, potential existence of genetically differentiated populations, and estimates of the level of admixture with closely related sympatric *Pseudemys* species. Many of these issues can be resolved with a range‐wide study to assess population connectivity, genetic diversity, and levels of admixture among sympatric populations, and to establish appropriate conservation units for this species and consequently identify priority areas for monitoring and protection. To date, genetic data on *P*. *alabamensis* have been collected on a relatively small sample size to clarify the taxonomic status of this species (Jackson et al., 2012; Spinks et al., 2013) or to assess genetic diversity at a single locality (Hieb et al., 2014). These studies found complex relationships between species in the *Pseudemys* genus, possibly originating from hybridization and introgression, and low genetic diversity for the Mobile‐Tensaw Delta population of *P*. *alabamensis* in Alabama.

Although hybridization has been observed within the *Pseudemys* genus, there are no documented cases of hybridization with *P*. *alabamensis* despite its co‐occurrence with two other *Pseudemys* species, *P*. *concinna* and *P*. *floridana*, which are known to hybridize in the area (Mount, 1975). In addition to observed hybridization of other *Pseudemys* species, we have also made anecdotal observations of mixed shell morphologies within *P*. *alabamensis* (N. Moreno, personal observation). Introgression with native *P*. *concinna* and *P*. *floridana*, or non‐native species that may have been introduced to the area, would have major conservation implications for *P*. *alabamensis*, as it would threaten the unique genetic identity of an already highly geographically restricted species with a likely low population size.

Here, we utilize mitochondrial DNA (mtDNA) and microsatellite markers to (1) identify the genetic structuring of populations of *P*. *alabamensis*, (2) measure intraspecific genetic diversity, (3) investigate the possibility of recent reductions in population sizes, and (4) assess potential hybridization with sympatric species. Our results can serve as a necessary basis to further develop conservation and management activities in collaboration with local conservation organizations and authorities and to raise awareness of the current status of imperilment of this species. As climate change increasingly impacts coastal populations, understanding the current distribution and genetic diversity of *P*. *alabamensis* will be critical for determining its long‐term survival potential, especially for small and isolated populations.

## 2 METHODS

### 2.1 Permits

This research was conducted under US Fish and Wildlife Service permit #TE40523A‐2, Mississippi Department of Wildlife, Fisheries, and Parks permit #0614181, and Alabama Fish and Wildlife permits #2018063278468680 and #2019097050868680. Trapping and handling methods were approved by the University of South Alabama Institutional Animal Care and Use Committee (IACUC Protocol No. 921991‐3).

### 2.2 Sample collection

Fieldwork was carried out from February to November in 2018–2019 throughout the range of *P*. *alabamensis* (Figure 2). Sampling was generally carried out one population at a time to reach a number of at least 10 individuals before moving to another location. However, if at least 10 individuals could not be sampled over several weeks, sampling was started at another locality. Independent of the number of individuals sampled during the first sampling effort, each population was then resampled at another time of the year to increase the number of individuals sampled per population. On any given sampling day/night, three to five traps were left at a site. Trapping of *Pseudemys* turtles was performed with encounter‐type aquatic hoop traps that were composed of an interior lead net and a double‐throated hoop trap attached at each end (paired net method). Hoop nets were 1.2 m in diameter and 4.6 m in length, while lead nets were 1.2 m in height and 9–12 m in length. Floats were added to hoop nets to maintain flotation and ensure access to air. Nets were anchored to the substrate with PVC tubing. Traps were left un‐baited and checked once every 36 h. Specific trap site selection was based on multiple factors: water depth, substrate, disturbance, basking logs, observed boat traffic, and submerged aquatic vegetation. In addition to trapping turtles, samples were also collected from roadkill individuals on the Mobile Bay Causeway (Figure 2), an area known for high rates of mortality for the species. Finally, an individual outside the recognized range of the species was sampled in Wolf River, Mississippi as well as two waif individuals from Dauphin Island, Alabama, and Gulfport, Mississippi. Waifs are stray individuals or individuals removed from their natural/typical habitat; in this case, waif refers to turtles that are presumably washed out of rivers. Sampled turtles were sexed on the basis of the length of the front claws, the cloaca position, and the thickness of the tail. In Emydidae (like *Pseudemys*), males have elongated foreclaws for titillation, a cloaca that is located outside the edge of the carapace, and thicker tails; females have a cloaca located closer to the edge of the carapace. Individuals that could not be confidently sexed were considered as juveniles. Sampled animals were also weighed with a scale and measured with a caliper for carapace and plastron width and length and shell height. The geographic locations of sampling sites were recorded with a handheld GPS. To prevent re‐sampling, turtles were marked for identification by notching the marginal scutes. Due to admixture between individuals of the cooter complex in the area (*P*. *concinna* and *P*. *floridana*), many individuals captured in this study presented mixed morphological characteristics; therefore, individuals were identified to the most similar species following morphological descriptions of the species in Alabama as in Mount (1975) and Leary et al. (2008). Briefly, *P*. *alabamensis* possesses an upper jaw with central notch flanked by a cusp on each side, complete eye bar, and a prefrontal arrow formed from the meeting of the sagittal head stripes with the supratemporal stripes. *Pseudemys concinna* possesses a smooth upper jaw, usually possessing a marked plastron and “C”‐ shaped marking on plural scutes, and lacking a complete eye bar. *Pseudemys floridana* has an unmarked plastron, unmarked undersides of posterior marginal scutes, a vertical bar on pleural scutes, and complete eye bars.

Blood for DNA extractions was collected from the subcarapacial sinus of each turtle. The skin of animals at the site was treated with 70% isopropyl alcohol prior to drawing blood. A maximum of 0.5% of body weight (max 2 ml of blood per turtle) was collected from each animal using a 23‐gauge needle and a 3‐ml syringe. All animals were released at the point of capture after blood sampling was performed and after ensuring that the puncture site was not bleeding and the animal was well. One ml of sampled blood was stored in 2‐ml microcentrifuge tubes with 1 ml of prepared blood preservative that consisted of 100 mM Tris–HCL, 100 mM EDTA, 10 mM NaCl, and 0.5% SDS. Samples were stored on ice until returned to the lab where they were then placed at −20°C for long‐term storage until DNA extractions were performed. DNA extractions were carried out using the Qiagen DNeasy Blood and Tissue kit (Qiagen, Inc., Valencia, CA) following the manufacturer's instructions for nucleated blood.

### 2.3 Mitochondrial DNA amplification and analysis

Fragments of the mitochondrial control region were amplified using the primers Des‐1 and Des‐2, which were originally developed by Starkey et al. (2003) for the painted turtle (*Chrysemys picta*). Twenty‐five μl reactions were prepared using 12.5 μl GoTaq G2 Green Master Mix (Promega), 0.5 μl 10 mg/ml bovine serum albumin, 1.2 μl each of 10 μM forward and reverse primers, 6.8 μl H_2_O, and 2.8 μl DNA extract. PCR conditions were as follows: 95°C for 3 min, 35 cycles of 95°C for 1 min, 55°C for 30 s, 72°C for 1 min; and a final 10‐min extension at 72°C. PCR products were checked on a 1% agarose gel to ensure proper amplification and then purified using ExoSAP‐IT (Applied Biosystems) according to the manufacturer's instructions. Sequencing was carried out by the DNA Analysis Facility at Yale University. Sequences were checked and manually edited using FinchTV (Treves, 2010). Cleaned sequences were aligned and collapsed into haplotypes using UGENE (Okonechnikov et al., 2012). Haplotypes were inputted into a BLAST (Basic Local Alignment Search Tool) search against the National Center for Biotechnology Information (NCBI) database. DnaSP (Rozas et al., 2017) was used to estimate haplotype diversity of all three species based on morphological assignment for each population. In order to visualize haplotype sharing between species, a parsimony haplotype network was created in PopART v1.7 (Leigh & Bryant, 2015) using the TCS method (Clement et al., 2000).

### 2.4 Microsatellite DNA amplification and analysis

Eight microsatellite loci were amplified in *P*. *alabamensis*, *P*. *concinna*, and *P*. *floridana*. These microsatellites were originally developed by King and Julian (2004) who isolated 30 microsatellite loci in *P*. *floridana*. Eight of these microsatellites were later shown to amplify successfully in *P*. *alabamensis* (Hieb et al., 2011) and were used in our study. Each locus was run separately in 25 μl reactions prepared using 5 μl 5× GoTaq Flexi buffer, GoTaq Flexi DNA Polymerase 5 μ/μl (Promega), 0.5 μl 25 mM dNTPs, 2 μl 25 mM MgCl_2_, 1.2 μl each of 10 μM forward and reverse primers, 11.98 μl H_2_O, and 3 μl DNA extract. Thermal cycler conditions for amplification of all eight microsatellite loci were as follows: 94°C for 2 min, 35 cycles of 94°C for 45 s, 58°C for 45 s, 72°C for 1 min, and a final 5 min extension at 72°C. Fragment analysis of amplified products was performed by the DNA Analysis Facility at Yale University. Fragment lengths were scored manually using Peak Scanner Software v2.0 (Applied Biosystems). Only a subset (*N* = 27) of *P*. *floridana* samples from Weeks Bay were amplified due to the high number of turtles caught (*N* = 68); all other *P*. *floridana* individuals sampled elsewhere were amplified (Table 1).

**TABLE 1:** Sampling effort and the number of individuals captured for each species across rivers

Sampled watershed	Sampling effort	*P. alabamensis*	# Individuals per effort	*P. concinna*	*P. floridana*
(1) Biloxi River	68	11 (8,1,2)	0.16	39 (19,10,10)	0
(2) Pascagoula River	50	18 (6,11,1)	0.36	7 (4,3,0)	0
(3) Bayou La Batre	10	1 (1,0,0)	0.10	3 (3,0,0)	1 (0,1,0)
(4) Fowl River	0.46	5 (2,2,1)	0.10	11 (6,5,0)	2 (1,1,0)
(5) Dog River	42	16 (7,9,0)	0.38	9 (5,4,0)	1 (0,1,0)
(6) Mobile‐Tensaw Delta^a^	52	24 (4,19,1)	0.46	32 (13,17,2)	1 (0,0,1)
(7) Weeks Bay	106	18 (4,14,0)	0.17	26 (16,10,0)	68 (23,45,0)
(8) Wolf River^b^	NA	1 (0,1,0)	–	0	0
(9) Waifs^b^	NA	2 (0,1,1)	–	0	0
Total	376	96		127	73

Note

Species identification was based on morphological assessment. Sampling effort is displayed as the number of “trap nights” where one trap is set for one night. Numbers next to sampled watersheds correspond to numbers on the map in Figure 2. Numbers of individuals for each sex are indicated in parentheses as (male, female, juvenile unsexed). Number of individuals per effort refers only to *P. alabamensis* captures.

^a^ This locality includes both roadkill and samples from live individuals collected in the water. The sampling effort for this locality refers only to samples obtained from live individuals collected in water.

^b^ Donated samples.

Null alleles and allelic dropout were checked within and across populations using MicroChecker (Van Oosterhout et al., 2004). Since null alleles can bias population structure analysis, FreeNA was used to calculate “uncorrected” and “corrected” (ENA correction, Chapuis & Estoup, 2007) pairwise *F*_ST_ values between river populations with *N* ≥ 5, between species, and between STRUCTURE identified clusters (see below). Allelic diversity, presence of private alleles, observed (*H*_O_) and expected (*H*_E_) heterozygosities, and inbreeding coefficient (*F*_IS_) were assessed with the software Genetix v4.05 (Belkhir et al., 2004). The program Fstat v2.9.4 was used to generate a sample size corrected allelic diversity (Goudet, 2003). Private alleles were considered for each population within each species (Petit et al., 1998) and for each species without distinguishing populations. ARLEQUIN v3.5.2.2 (Excoffier & Lischer, 2010) was used to calculate the significance of *F*_ST_ values, linkage disequilibrium between loci across all populations, and departure from Hardy‐Weinberg equilibrium. BOTTLENECK v1.2.02 (Cornuet & Luikart, 1996) was used under all three mutational models available to detect signatures of historic bottlenecks within populations. The program Ne ESTIMATOR was used to infer breeding population size estimates for each river population (Do et al., 2014).

The program STRUCTURE v2.3.4 (Pritchard et al., 2000) was used to identify patterns of genetic structure of *P*. *alabamensis* across the study area. The correlated allele frequency model with admixture was used to examine all *Pseudemys* captured as a whole, *P*. *alabamensis* alone, and *P*. *concinna* alone. *Pseudemys floridana* was not run independently of the other species due to only a few individuals being found outside of the Weeks Bay system (Table 1). STRUCTURE analysis consisted of 10 independent runs for each *K* value (1–10) with a burn‐in period of 100,000 followed by an additional 100,000 repetitions. In order to determine the best value of K (number of clusters) for each species, the Δ*K* statistic (Evanno et al., 2005) was calculated using STRUCTURE HARVESTER (Earl, 2012). STRUCTURE was also used to calculate the estimated membership coefficients *Q* for each individual in each cluster. *Q* indicates whether each individual belongs to one or, if admixed, to several clusters. Finally, a principal component analysis (PCA) was used to further assess the level of introgression based on microsatellite data using the software Genetix v4.05 (Belkhir et al., 2004).

## 3 RESULTS

**FIGURE 3:**
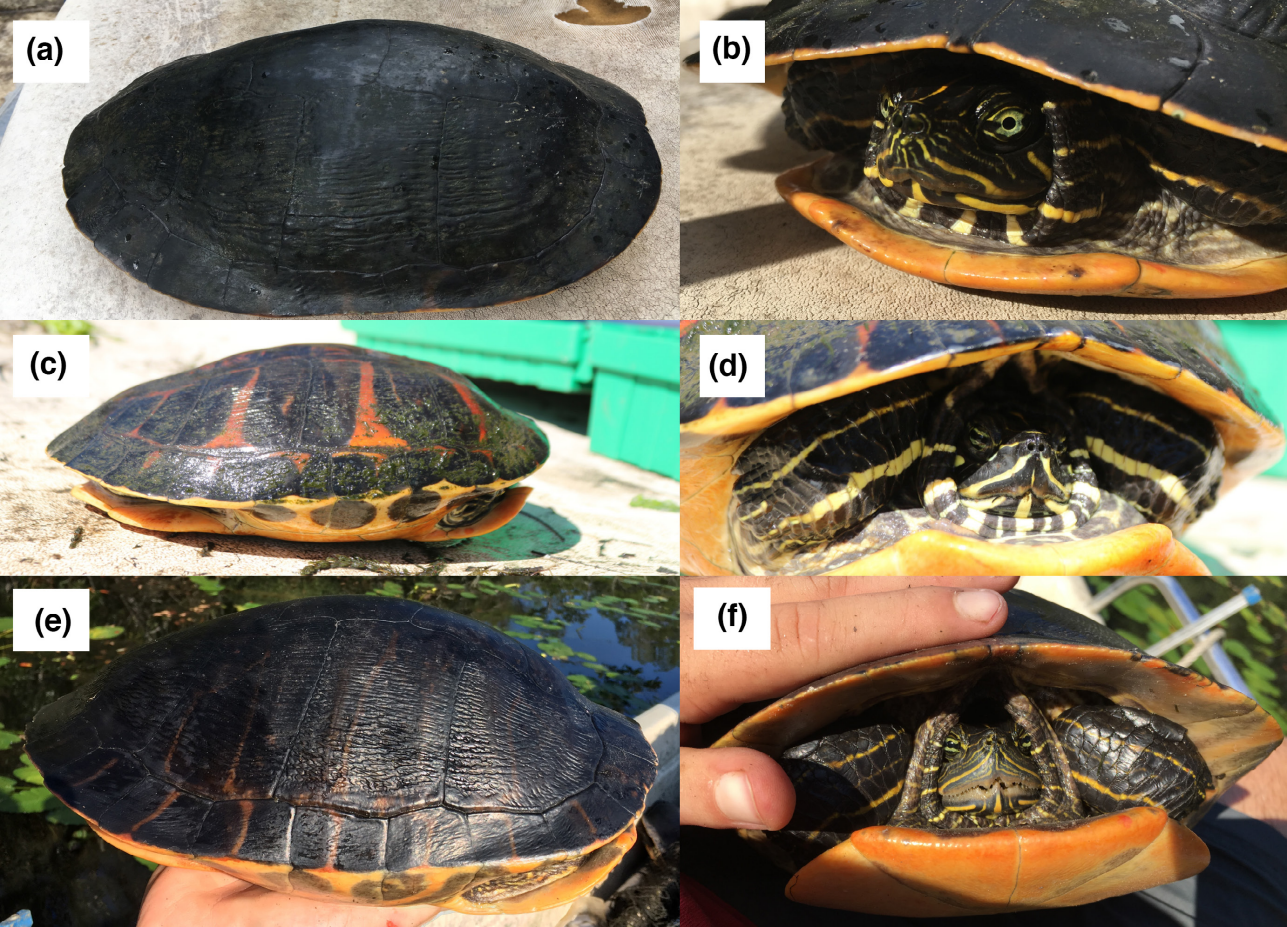
Photos illustrating two captured individual turtles that were considered to be potential hybrids based on morphological characteristics. (a, b) Individual identified as *P*. *alabamensis* *x* *P*. *concinna* hybrid due to strongly reduced jaw cusp, incomplete eye bars, and incomplete prefrontal arrow. Individual was found in Bayou La Batre, Mobile County, Alabama. (c, d) Individual considered to be *P*. *alabamensis* *x* *P*. *peninsularis* hybrid due to resemblance to *P*. *peninsularis* and presence of *P*. *alabamensis* characteristics. Individual found in Dog River, Mobile County, Alabama. (e, f) Typical *P*. *alabamensis* individual with no morphological characteristics indicating hybridization

In total, 296 *Pseudemys* turtles were captured from water bodies known to be inhabited by *P*. *alabamensis* (Table 1). 96, 127, and 73 of these individuals were morphologically identified as *P*. *alabamensis*, *P*. *concinna*, and *P*. *floridana*, respectively. Despite many attempts, capture rates of *P*. *alabamensis* for some localities (e.g., Fowl River and Biloxi River) were low (Table 1). One *P*. *alabamensis* individual was found in Wolf River, Mississippi, which is outside the currently recognized range of this species. Two potential hybrids between *P*. *alabamensis* and other *Pseudemys* species were identified in the field on the basis of morphological characteristics (Figure 3). One of these individuals, caught in Bayou La Batre, Alabama, appeared to be a *P*. *alabamensis × P*. *concinna* hybrid based on multiple morphological features including a strongly reduced jaw cusp, incomplete eye bars, and incomplete prefrontal arrow formed from the meeting of the sagittal head stripes with the supratemporal stripes. The other potential hybrid, captured in Dog River, resembled *P*. *peninsularis*, a non‐native species, but still possessed identifying characteristics of *P*. *alabamensis*. *P*. *alabamensis* and *P*. *concinna* co‐occurred in all the sampled rivers, while *P*. *floridana* mostly co‐occurred with these other two species in Weeks Bay (Table 1).

Samples of *P*. *alabamensis* from the Biloxi River showed a skew toward males (Table 1). However, despite the lack of sampled females, this is still a breeding population as shown by an abundance of hatchling *P*. *alabamensis* that were observed in the area at the time of sampling (N. Moreno, personal observation). For the Mobile‐Tensaw Delta, our sampling included more females than males, as a large portion of our samples for this area came from road‐kill individuals, which affects female turtles more than males (Marchand & Litvaitis, 2004; Steen & Gibbs, 2004). Overall, for *P*. *concinna*, more males than females were captured at all sites, except for the Mobile‐Tensaw Delta.

### 3.1 Mitochondrial DNA analysis

**FIGURE 4:**
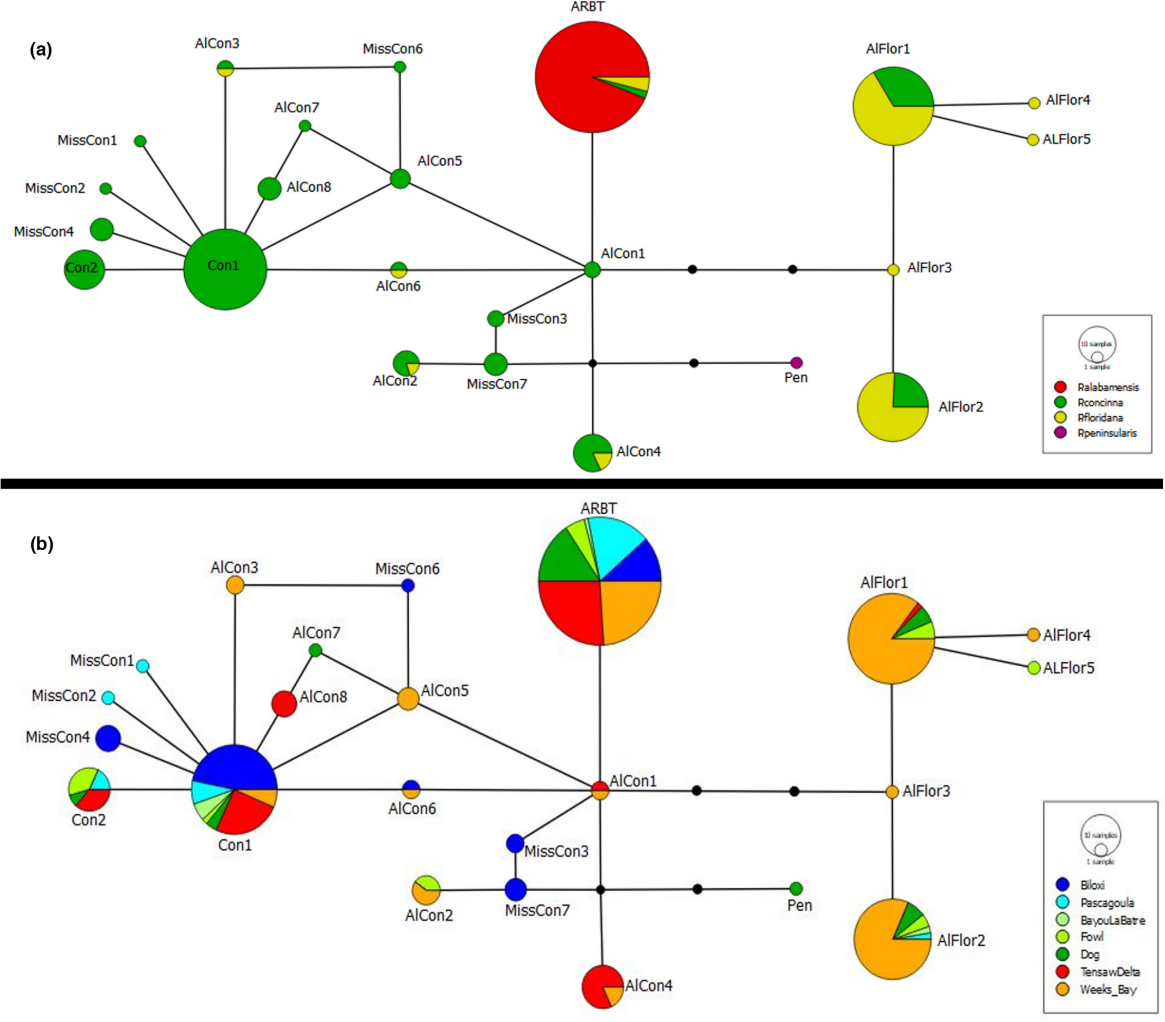
Haplotype networks based on mitochondrial control region sequence data. (a) Haplotype network showing the connectivity and haplotype sharing among species. (b) Haplotype network showing haplotype distribution and sharing among sampling localities

A 587 bp fragment of the mtDNA control region was amplified from all 296 *Pseudemys* turtles sampled. Only 2 haplotypes were identified for *P*. *alabamensis*: one haplotype (ARBT) was common among all sampled populations, while the other (Pen) was present only in a single individual from Dog River (Table 2, Figure 4). BLAST search confirmed the common ARBT haplotype to be *P*. *alabamensis*, which was identical to a previously found haplotype (Jackson et al., 2012) (GenBank: GQ395751). The individual from Dog River with the Pen haplotype exhibited mixed morphological characteristics. This haplotype is four mutational steps from the ARBT *P*. *alabamensis* haplotype and matched *P*. *peninsularis* (GenBank: KC687235), a species that is normally only found on the Florida peninsula. Out of 200 samples of *P*. *concinna* and *P*. *floridana*, 19 variable nucleotide positions, including one insertion found in two individuals from Biloxi, Mississippi (haplotype = MissCon7), were identified, defining 22 haplotypes. The ARBT haplotype of *P*. *alabamensis* was found in four individuals of *P*. *floridana* and two of *P*. *concinna*. Twelve haplotypes were unique to individuals morphologically identified as *P*. *concinna* (Con1, Con2, AlCon1, MissCon3, MissCon7, AlCon5, MissCon6, AlCon7, AlCon8, MissCon1, MissCon2, MissCon4), three haplotypes were unique to individuals morphologically identified as *P*. *floridana* (AlFlor3, AlFlor4, AlFlor5), and six haplotypes were shared between *P*. *concinna* and *P*. *floridana* (AlCon2, AlCon3, AlCon4, AlCon6, AlFlor1, AlFlor2) (Figure 4). Of the 30 individuals with shared haplotypes between species, 25 individuals morphologically identified as *P*. *concinna* clustered with mostly *P*. *floridana* haplotypes and five individuals morphologically identified as *P*. *floridana* clustered with mostly *P*. *concinna* haplotypes. The star organization of the 12 haplotypes unique to *P*. *concinna* suggests a population expansion from the most represented haplotype (Con1) for this species. Haplotype diversity for *P*. *concinna* averaged 0.76 (range 0.53–0.86 among populations) and was 0.64 in the *P*. *floridana* Weeks Bay population (Table 2), which is the only population of this species with *N* > 5. Haplotype sequences have been deposited in NCBI GenBank (see Data Availability section for accession numbers).

**TABLE 2:** Sample sizes and genetic diversity for each population of each species for the mitochondrial control region marker (mtDNA)

Species	Sampled watershed	*N* (mtDNA)	Number mtDNA haplotypes	mtDNA haplotype diversity
*P. alabamensis*	Weeks Bay	18	1	0
	Mobile‐Tensaw Delta	24	1	0
	Dog River	16	2	0.125
	Fowl River	5	1	0
	Pascagoula River Delta	17	1	0
	Biloxi River	11	1	0
*P. concinna*	Weeks Bay	26	9	0.837
	Mobile‐Tensaw Delta	32	7	0.778
	Dog River	9	5	0.861
	Fowl River	11	5	0.818
	Pascagoula River Delta	7	4	0.714
	Biloxi River	37	6	0.53
*P. floridana*	Weeks Bay	68	9	0.637

### 3.2 Microsatellite

The eight microsatellite loci analyzed were polymorphic in all species and populations, with the exception of one locus (D87) in one population (Fowl River) for *P*. *alabamensis*, and two loci (B91 in Pascagoula and D55 in Pascagoula and Fowl River) for *P*. *concinna*. The eight loci ranged between 4‐10 alleles each for *P*. *alabamensis*, between 4‐17 alleles each for *P*. *concinna*, and between 3‐12 alleles each for *P*. *floridana*. In *P*. *alabamensis*, private alleles were found exclusively in the Mobile Bay populations, with the Mobile‐Tensaw Delta possessing four private alleles, Weeks Bay three private alleles, and Dog River two private alleles (Table 3). In *P*. *concinna*, private alleles were found in all but the Fowl River population with Weeks Bay and Biloxi River possessing the most private alleles (nine and five, respectively) (Table 3). For each species, *P*. *alabamensis* had 14 private alleles in total, *P*. *concinna* had 20, and *P*. *floridana* had 5.

**TABLE 3:** Sample sizes and genetic diversity indices for each population (with *N* ≥ 5) of each species for microsatellite data

Species	Location	*N*	*N*_A_	*N*_U_	*H*_e_	*H*_o_	*A*/*A*_corr_	*F*_IS_
*P. alabamensis*	Biloxi River	11	24	–	0.55	0.45	3.38/2.93	0.222 (0.015–0.301)
	Pascagoula River	17	26	–	0.47	0.39	3.63/2.69	0.194 (0.038–0.285)
	Fowl River	5	20	–	0.45	0.35	2.88/2.88	0.329 (−0.12–0.381)
	Dog River	16	32	2	0.55	0.48	4.63/3.41	0.157 (−0.011–0.254)
	Mobile Tensaw Delta	24	33	4	0.55	0.45	4.75/3.34	0.198 (0.067–0.279)
	Weeks Bay	18	30	3	0.54	0.47	4.37/3.34	0.147 (−0.031–0.257)
*P. concinna*	Biloxi River	38	40	5	0.57	0.40	5/3.73	0.312 (0.218–0.376)
	Pascagoula River	7	24	1	0.45	0.45	3/3.0	0.074 (−0.20–0.133)
	Fowl River	11	31	–	0.47	0.42	3.88/3.43	0.112 (−0.119–0.204)
	Dog River	9	33	2	0.50	0.42	4.13/3.80	0.224 (−0.033–0.317)
	Mobile Tensaw Delta	20	42	4	0.56	0.46	5.25/4.0	0.206 (0.053–0.30)
	Weeks Bay	14	35	9	0.47	0.38	4.38/3.63	0.216 (0.057–0.277)
*P. floridana*	Weeks Bay	27	46	–	0.51	0.41	5.75/6.17	0.122 (−0.002–0.21)

Note

*N* = sample size, *N*_A_ = number of alleles, *N*_U_ = number of private alleles, *H*_O_ and *H*_E_ = observed and expected heterozygosity, respectively, *A* = allelic diversity (Average number of alleles/locus), *A*_corr_ = allelic diversity corrected for sample size, and *F*_IS_ = inbreeding coefficient.

Two loci, B21 and D79, showed evidence of null alleles in all three species for half or more of the sampled populations, with the Biloxi population especially affected by the presence of null alleles in *P*. *concinna*. Overall, null alleles were identified in 23 of the 96 combinations of loci × populations × species (8 loci, 6 populations, 2 species). F‐tests run on corrected and uncorrected *F*_ST_ values obtained using FreeNA indicated that the presence of null alleles does not affect *F*_ST_ estimates (*p* > .05 for each species comparison). Therefore, all microsatellite loci were used in subsequent analyses. No loci showed significant linkage disequilibrium (*p* < .01) across populations providing evidence of independent segregation of loci used. All populations of *P*. *alabamensis* and all but one *P*. *concinna* population showed departure from Hardy‐Weinberg Equilibrium at the locus D79, most likely as a result of the null allele and higher homozygosity levels. Among all populations, *P*. *concinna* from Biloxi possessed the most significant departures at five of the eight loci, with lower‐than‐expected heterozygosity. Similarly, the single *P*. *floridana* population with *N* > 5 displayed significant departure from Hardy‐Weinberg equilibrium at three loci. Within species, *F*_ST_ values among populations ranged between 0 and 0.28 and between 0 and 0.19 for *P*. *alabamensis* and *P*. *concinna*, respectively. Populations from Pascagoula (Mississippi) for both *P*. *alabamensis* and *P*. *concinna* were the most distinct (*F*_ST_ values > 0.17) from their counterpart populations in Alabama (Table 5). Pairwise *F*_ST_ values calculated for *P*. *alabamensis* vs. *P*. *concinna* and *P*. *floridana* were 0.106 and 0.132, respectively, while Fst between *P*. *concinna* and *P*. *floridana* was found to be low (0.065), likely as a result of admixture between the two species.

Genetic diversity, like allelic diversity and heterozygosity, was generally low. Allelic diversity (A) in populations with *N* ≥ 5 ranged from low (2.88) to moderate (4.75) in *P*. *alabamensis* (mean 3.94), from 3 to 5 (mean 4.27) in *P*. *concinna*, and was relatively higher (5.75) in the single *P*. *floridana* population found in Weeks Bay (Table 3). Observed heterozygosity (*H*_O_) ranged from 0.35 to 0.48 in *P*. *alabamensis*, from 0.38 to 0.45 in *P*. *concinna*, and was 0.41 in *P*. *floridana*. Expected heterozygosity (*H*_E_) ranged from 0.45 to 0.55 in *P*. *alabamensis*, from 0.45 to 0.57 in *P*. *concinna*, and was 0.51 in *P*. *floridana*. All populations for all species, except for *P*. *concinna* in Pascagoula, showed an excess of homozygosity with H_O_ having much lower values than *H*_E_. In *P*. *alabamensis*, bottleneck analysis identified one significant occurrence (*p* < .05) for the Pascagoula population (*N* = 17) under the Stepwise Mutation Model. *N*_e_ estimates showed support for low breeding population sizes (*N*_e_ < 30) in the Biloxi River and Fowl River populations of *P*. *alabamensis* (Table 4). Consequently, inbreeding was observed for these two populations with *F*_IS_ values of 0.22 and 0.33, respectively (Table 3). Ne ESTIMATOR found little evidence of low breeding population sizes in *P*. *concinna* or *P*. *floridana* (Table 4).

**TABLE 4:** Breeding population size as estimated by NeESTIMATOR

River	*P. alabamensis*	*P. concinna*	*P. floridana*
Weeks Bay	Infinite	Infinite	112.1 – Infinite
Mobile‐Tensaw Delta	97.5–218.6	Infinite	–
Dog River	170.3 – Infinite	Infinite	–
Fowl River	19.5	101.4 – Infinite	–
Pascagoula River	66.5 – Infinite	Infinite	–
Biloxi River	20.8–26.7	48.1 – Infinite	–

**FIGURE 5:**
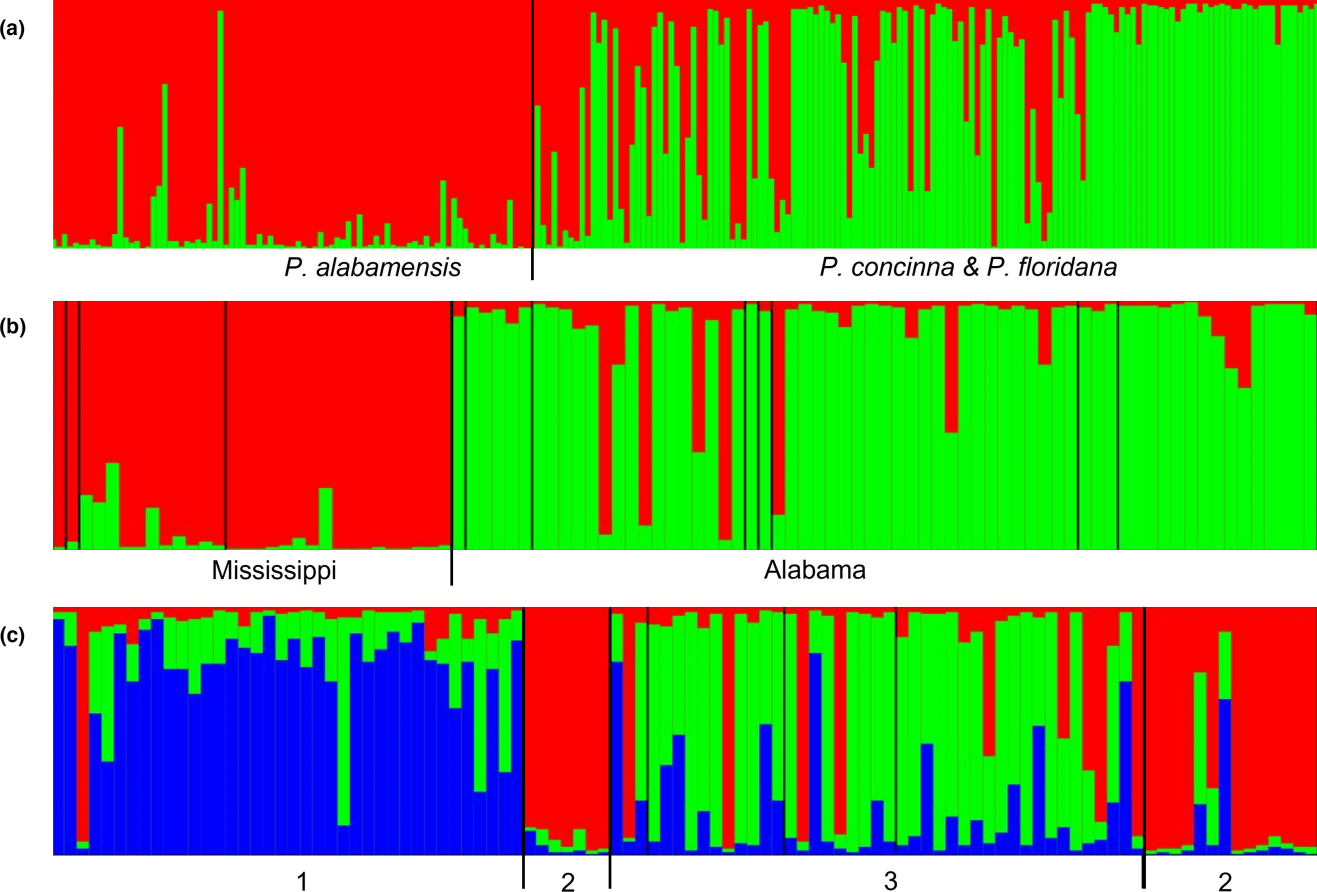
STRUCTURE graphs based on eight microsatellite loci. (a) STRUCTURE graph of all turtles studied showing clusters of *P*. *alabamensis* and the two sympatric cooter species. (b) STRUCTURE graph of *P*. *alabamensis* showing Mississippi and Alabama clusters. (c) STRUCTURE graph of *P*. *concinna*. Subdivisions of *P*. *concinna* structure graph as follows: 1. Biloxi population, 2. Pascagoula and Weeks Bay population, 3. Populations from Mobile County, Alabama

STRUCTURE analysis of all *Pseudemys* species considered in this work identified an optimum clustering of *K* = 2 with evidence of some admixture (Figure 5). The two clusters corresponded to *P*. *alabamensis* and *P*. *concinna*/*P*. *floridana*, respectively. When the clustering analysis was performed only on *P*. *alabamensis*, optimum clustering was also *K* = 2 corresponding to Mississippi and Alabama populations. The analysis repeated only on *P*. *concinna* found an optimum clustering level of *K* = 3 corresponding to (1) Biloxi River, (2) Fowl River, Dog River, and Mobile Tensaw Delta, and (3) Pascagoula River and Weeks Bay populations. *F*_ST_ of *P*. *concinna* clusters was generally low, and follows the cluster numbers listed above: cluster 1 vs. 2 = 0.043, cluster 1 vs. 3 = 0.078, cluster 2 vs. 3 = 0.072. All microsatellite allele scoring information is provided in Supplementary Data 1 on Dryad.

### 3.3 Hybridization

Of the 96 samples morphologically identified as *P*. *alabamensis*, two individuals were considered to be potential hybrids based on mixed morphological characteristics and the presence of reduced *P*. *alabamensis* identifying characteristics. One of these individuals from Dog River possessed a *P*. *peninsularis* mtDNA haplotype, seven microsatellite loci with alleles matching *P*. *alabamensis*, and one microsatellite locus possessing an allele not found in any other individual or species studied here. The other individual (from Bayou La Batre), despite having the *P*. *alabamensis* mtDNA haplotype (ARBT), clustered with the cooter species (*P*. *concinna* and *P*. *floridana*) in the STRUCTURE analysis based on microsatellite loci. Of the 102 *P*. *concinna* and 30 *P*. *floridana* individuals with both mtDNA data and microsatellite data, 27 (26.5%) *P*. *concinna* and 6 (20%) *P*. *floridana* individuals possessed conflicting species assignments between the two marker types. Two morphologically identified *P*. *concinna* and four *P*. *floridana* individuals possessed the *P*. *alabamensis* haplotype (ARBT), although the *P*. *floridana* individuals did not group with *P*. *alabamensis* in the STRUCTURE analysis based on microsatellites. One of these four individuals possessed a bright red plastron, a characteristic not present in *P*. *floridana*, which typically possess plain yellow plastrons; however no other potential *P*. *alabamensis* morphological characteristics were seen in these six individuals. Five of the six cooters that displayed the *P*. *alabamensis* haplotype were found in the rivers of Weeks Bay, while the sixth was found in the Mobile Tensaw Delta.

Based on microsatellite data, *F*_ST_ values between *P*. *alabamensis* and each of the other two sympatric *Pseudemys* species were lower (0.106 and 0.132 for *P*. *concinna* and *P*. *floridana*, respectively) than *F*_ST_ values observed between *P*. *alabamensis* from Pascagoula versus the populations in Alabama (*F*_ST_ ranging from 0.17 to 0.28; Table 5), further supporting the occurrence of hybridization between species. Between *P*. *alabamensis* and *P*. *concinna*, three of the alleles private to populations within a single species were found in the other species. Sharing of private alleles may be an indication of admixture and introgression. One private allele from locus B91 that was only found in the Dog River population of *P*. *alabamensis* was also found in the Biloxi River population of *P*. *concinna* (frequency of the allele in Biloxi = 0.026). One private allele from locus D121 that was only found in the Weeks Bay population of *P*. *alabamensis* was also found to be a common allele in *P*. *concinna* (frequency of the allele in *P*. *concinna* reached 0.278 in the Mobile‐Tensaw Delta population). One allele from locus D28 that was a private allele in the Biloxi *P*. *concinna* population was also found in the neighboring Pascagoula population of *P*. *alabamensis* (frequency of the allele in *P*. *alabamensis* in Pascagoula = 0.027).

**TABLE 5:** *F*_ST_ pairwise values based on microsatellite data for populations of *P*. *alabamensis* (bottom left), and *P*. *concinna* on the top right axis

	Weeks Bay	Mobile‐Tensaw Delta	Dog River	Fowl River	Pascagoula River	Biloxi River
Weeks Bay	–	.**103**	.**110**	.**123**	.**186**	.**102**
Mobile‐Tensaw	.**025**	–	.**035**	.021	.**147**	.**046**
Dog River	.**027**	.000	–	.001	.**148**	.**060**
Fowl River	.**145**	.**093**	.**068**	–	.**193**	.**045**
Pascagoula River	.**222**	.**225**	.**172**	.**282**	–	.**138**
Biloxi River	.**100**	.**077**	.**046**	.**125**	.**095**	–

Note

Bold values are significant at *p* < .05.

Hybridization appears to occur at a higher rate between *P*. *concinna* and *P*. *floridana*. *F*_*ST*_ between the two species was 0.065, much lower than between *P*. *alabamensis* and either of these two species and even within *P*. *alabamensis*. The *P*. *floridana* population with >5 individuals possessed 6 of the alleles that were private alleles within *P*. *concinna* populations and 1 allele that was considered a private allele within a *P*. *alabamensis* population.

**FIGURE 6:**
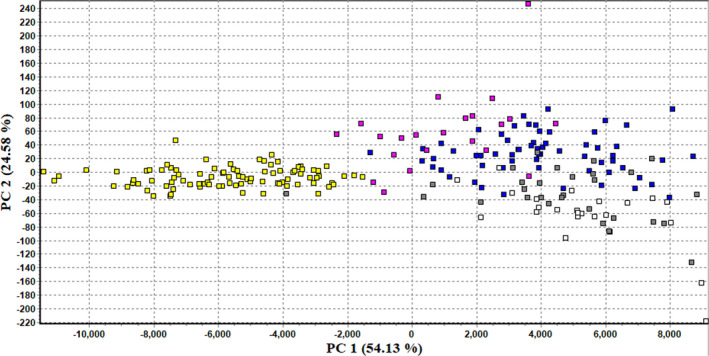
Principal Component Analysis (PCA) based on microsatellite and mtDNA data. Shown are PC1 and PC2. Distinct colors refer to different species or admixed individuals. Yellow: *P*. *alabamensis*, Blue: *P*. *concinna*, White: *P*. *floridana*, Pink: admixed individuals between *P*. *alabamensis* and *P*. *concinna*/*P*. *floridana*, Gray: admixed individuals between *P*. *concinna* and *P*. *floridana*

When examining species assignment and admixture by STRUCTURE, we found that three individuals (3%) that were morphologically identified as *P*. *alabamensis* were assigned to *P*. *concinna* (one from Bayou La Batre with *Q* = 0.95 was assigned to *P*. *concinna*/*P*. *floridana*, one from Fowl River with *Q* = 0.68, and one from Weeks Bay with *Q* = 0.66 to *P*. *concinna* and 0.77 to *P*. *floridana*). Another individual from Pascagoula morphologically identified as *P*. *alabamensis* showed admixture with mixed assignment between *P*. *alabamensis* and *P*. *concinna* (*Q* = 0.62). Signs of hybridization with *P*. *alabamensis* were also found in individuals morphologically identified as *P*. *concinna*. Of the 102 individuals morphologically identified as *P*. *concinna*, 12 individuals (12%) were assigned to *P*. *alabamensis* with *Q* ≥ 0.7, and another 7 (23%) showed mixed assignments (0.5 < *Q* < 0.7) between the two species. Across all the populations, the Biloxi river was the locality where many individuals (8 out of 38 with *Q* ≥ 0.7) morphologically identified as *P*. *concinna* were assigned to *P*. *alabamensis* on the basis of microsatellite data. We also found that two individuals out of 30 (7%) that were morphologically identified as *P*. *floridana* showed evidence of admixture (*Q* ≥ 0.7). Finally, of all individuals of *P*. *concinna* and *P*. *floridana*, 33 out of 103 (32%) *P*. *concinna* were either assigned to *P*. *floridana* (*Q* > 0.7) or showed admixture (0.5 < *Q* < 0.7), and 5 out of 30 (17%) *P*. *floridana* were also either assigned to *P*. *concinna* (*Q* ≥ 0.7) or showed admixture (all *Q* values in Supplementary Data 2 on Dryad). Finally, PCA run on microsatellite and mtDNA data indicates a clear distinction of *P*. *alabamensis* individuals (in yellow in Figure 6) from *P*. *concinna* (blue) and *P*. *floridana* (white) along PCA1. The individuals showing admixture between *P*. *alabamensis* and *P*. *concinna*/*P*. *floridana* (pink) were placed in between the two main groups and grouping more towards *P*. *concinna*/*P*. *floridana*, suggesting the presence of both F1 hybrids and backcrosses of F1 hybrids with *P*. *concinna*/*P*. *floridana* individuals (Figure 6).

## 4 DISCUSSION

In this study, we assessed genetic diversity, population structure, and potential hybridization of the endangered *P*. *alabamensis* and co‐occurring congeneric species. While previous studies have also addressed some of these questions (Hieb et al., 2014; Jackson et al., 2012), the sample sizes, distribution range of sampled populations, and/or genetic markers were limited. In our study, we used both mitochondrial and microsatellite markers to analyze *P*. *alabamensis* from seven rivers throughout the entire narrow range of this species. Using mitochondrial DNA, we found no genetic differentiation within or among populations of *P*. *alabamensis* due to a complete lack of mtDNA variation. Low levels of mitochondrial diversity are not uncommon in turtles that are of conservation concern (Rosenbaum et al., 2007; Vargas‐Ramírez et al., 2007). However, different than what has been observed in other endangered species, only one haplotype was found across 96 individuals from the entire distribution range of *P*. *alabamensis*. A comparable lack of mitochondrial diversity to *P*. *alabamensi* *s* has been noted in a related species, *Pseudemys gorzugi* (Bailey et al., 2008). *Pseudemys gorzugi* also inhabits a restricted range, although larger than *P*. *alabamensis*, being found only in the Rio Grande and Pecos Rivers in North America. *Pseudemys alabamensis* displayed no genetic variation across populations in the mtDNA control region; conversely, *P*. *concinna* and *P*. *floridana* showed a higher degree of genetic variation within and among populations. This may be indicative of the larger population sizes of these other species and may reflect their greater overall distribution range compared to *P*. *alabamensi* *s*. *Pseudemys concinna* populations in the area are likely connected to greater populations occurring in northern Alabama and Mississippi through the larger rivers of the Mobile‐Tensaw Delta and Pascagoula Delta watersheds. Individuals dispersing from the northern populations may contribute to the genetic variation of these smaller isolated coastal populations.

Microsatellite data also indicate low genetic diversity for *P*. *alabamensis* with overall lower allelic diversity than the other two sympatric congenerics and lower than expected heterozygosity. Signs of inbreeding were observed in two populations: Fowl River and Biloxi River. Biloxi showed signs of inbreeding also for *P*. *concinna*, most likely the result of low population sizes for both species at this site (the estimated breeding population for *P*. *alabamensis* at Biloxi was in fact low; see also hybridization discussion below). Despite the overall low genetic diversity observed in *P*. *alabamensis*, microsatellite data support genetic differentiation between Mississippi and Alabama populations of this species, in agreement with slight morphological differences previously observed between these areas (Leary et al., 2003). Although this genetic structure and morphological differentiation may be the result of genetic drift, little gene flow between Mississippi and Alabama populations may occur due to the large distance between the mouth of the Pascagoula River Delta and the Alabama populations. Land and saltwater can hinder gene flow for freshwater species that are distributed in riverine systems across the Gulf of Mexico (Soltis et al., 2006), including the Pascagoula River (e.g., Dugo et al., 2004; Ennen et al., 2010).

We found no structure among populations of *P*. *alabamensis* in the Mobile Bay (populations 3–7 in Figure 2). The presence of multiple alleles that are found in all major Alabama populations, but not in Mississippi populations, also suggests the occurrence of gene flow among the Alabama populations. This may be due to the potential migration of individuals between these populations due to the lower salinity of the Mobile Bay compared to the Mississippi Sound. Movement of individuals across populations, including towards the lower part of the Mobile‐Tensaw Delta, may be permitted by the fact that *Pseudemys* species have been reported to possess some level of tolerance to brackish water (Agha et al., 2018). This is further supported by the presence of barnacles on the shells of some individuals in our study indicating exposure to higher salinity waters (N. Moreno, personal observation).

We observed admixture among the three species. Individuals of *P*. *concinna* and *P*. *floridana* in the region can be difficult to tell apart due to hybridization between the two (Mount, 1975; Spinks et al., 2013). We found haplotype sharing and mixed assignments between species based on microsatellite data, even for individuals which could be confidently assigned to a species based on morphological characteristics. Specifically, based on microsatellite data, around 50% of the individuals that were morphologically identified as *P*. *concinna* or *P*. *floridana* were assigned to the other species based on genetics. Haplotype sharing was also seen to a lesser degree (five individuals) between *P*. *alabamensis* and *P*. *concinna*/*P*. *floridana* individuals and was confirmed by microsatellite data (more than 40% of individuals showed admixture between *P*. *alabamensis* and *P*. *concinna*/*P*. *floridana* based on microsatellite data). To our knowledge, these data represent the first published evidence of hybridization between *P*. *alabamensis* and sympatric *Pseudemys* species. In all of these cases of haplotype sharing, animals were morphologically identified as *P*. *concinna* or *P*. *floridana*, but had the *P*. *alabamensis* mtDNA haplotype, suggesting that hybridization in *P*. *alabamensis* may be largely driven by males of *P*. *concinna* and *P*. *floridana* breeding with female *P*. *alabamensis*. In Weeks Bay and in the Mobile‐Tensaw Delta, where we found instances of haplotype sharing among species, we sampled an excess of female versus male *P*. *alabamensis* (Table 1). Hybridization of *P*. *alabamensis* with congeneric species across its distribution range may overall be driven by decreased opportunities to find mates of the same species (“desperation hypothesis”, Hubbs, 1955). This may be the result of a potentially skewed sex ratio in Weeks Bay and Mobile‐Tensaw Delta populations and low breeding population sizes in Biloxi, Bayou La Batre, and Fowl River (Tables 1, 3, and 4). In Biloxi, for example, we found many individuals morphologically identified as *P*. *concinna*, but genetically assigned to *P*. *alabamensis*. In Bayou La Batre, we also found a single specimen morphologically identified as *P*. *alabamensis* possessing a strongly reduced red belly (a characteristic of *P*. *alabamensis*); this individual grouped with *P*. *concinna and P*. *floridana* in STRUCTURE, but had the mtDNA haplotype of *P*. *alabamensis*, suggesting a possible *P*. *concinna × P*. *alabamensis* hybrid origin. Furthermore, our data point to the presence of both F1 hybrids between *P*. *alabamensis* and *P*. *concinna*/*P*. *floridana* and backcrosses of F1 hybrids with *P*. *concinna*/*P*. *floridana* individuals. This indicates not only that the hybrids are viable and able to reproduce, but that backcrosses of F1 hybrids occur only with individuals of *P*. *concinna*/*P*. *floridana*, further supporting the hypothesis that hybridization may be driven by lower population sizes in *P*. *alabamensis*. Future studies using more loci can better assess the level of genomic introgression among these co‐occurring species. Finally, based on mtDNA data, we found one female in Dog River that was morphologically identified as *P*. *alabamensis* that possessed a *P*. *peninsularis* haplotype. The home range of *P*. *peninsularis* is isolated to the Florida peninsula and is not native to the range of *P*. *alabamensis*. It is possible that this individual represents a *P*. *peninsularis × P*. *alabamensis* hybrid offspring of a female *P*. *peninsularis* that was released into Dog River and bred with native *P*. *alabamensis*.

Overall, based on our results, *P*. *alabamensis* is experiencing significant admixture with congeneric co‐occurring species across its entire restricted distribution range. Hybridization is a well‐known phenomenon in species of conservation concern with limited population sizes (e.g., see Chattopadhyay et al., 2019 and references therein), and it presents a challenge for conservation management (Allendorf et al., 2005; Mallet, 2005; Wayne & Shaffer, 2016). Although historically hybridization has generally been seen as a threat to endangered species, hybridization can also be a component of evolutionary processes and the origin of new species (Draper et al., 2021; Haig & Allendorf, 2006; Willis, 2020). In the US, the Endangered Species Act (ESA) provides guidelines that can be interpreted by the US Fish and Wildlife Service, depending, for example, on the origin and ecological role of the hybrid species and whether or not hybrids can be used for recovery of endangered parental species (Haig & Allendorf, 2006; Willis, 2020). Based on this, protection of hybrids could be possible, although it is generally discouraged. Mostly, removal of hybrids is suggested when hybridization presents a threat to an endangered species (Draper et al., 2021; Haig & Allendorf, 2006), as in the case of *P. alabamensis*. Our study represents the first genetic study supporting the endangered status of *P*. *alabamensis* throughout its range and provides evidence that the Mississippi and Alabama populations of *P*. *alabamensis* are genetically distinct.

The overall low amount of genetic diversity observed at mitochondrial and nuclear (microsatellite) levels in *P*. *alabamensis*, the severely limited geographic range of this species, and the occurrence of hybridization throughout its distribution require the urgent development of targeted conservation actions. It has been shown that low genetic diversity and inbreeding in combination with an endemic restricted distribution may make species more susceptible to diseases and to the risk of genetic swamping due to hybridization (Georges et al., 2018). If hybrids could be identified with confidence based on morphological characteristics, targeted removal of hybrids could help avoid hybrid swamping of *P*. *alabamensis*. Although two individuals were morphologically identified as hybrids of *P*. *alabamensis* and confirmed as such by genetic data, not all genetically identified hybrids were easily identified by morphological characteristics. As morphological identification of hybrids and closely related species may be challenging and sometimes misleading (Chiari & Claude, 2012), further efforts should be made to develop methods that could be applied in the field to identify hybrids with confidence in order to remove them.

Our results also identify several *P*. *alabamensis* populations of higher conservation concern due to their low population sizes and consequent inbreeding and hybridization: Bayou La Batre, Biloxi, Weeks Bay, and Fowl River. Furthermore, considering the observed genetic distinction of populations from Alabama and Mississippi, specific management actions should be developed to preserve their uniqueness. This includes searching for additional unknown branches of these main riverine systems where the species could occur. Finally, climate change is predicted to strongly affect coastal areas and wetlands in the Gulf of Mexico (Anderson et al., 2014; Mulholland et al., 1997; Scavia et al., 2002), influencing the salinity of coastal watersheds and consequently their vegetation, and potentially changing the connectivity of existing watersheds due to sea‐level rise. These factors can greatly influence the geographic range of species (Garroway et al., 2010). Thus, the imperiled status of *P*. *alabamensis* could further worsen due to changes in habitat salinity, effects on its vegetation food sources, and potentially increased hybridization. It is therefore imperative that measures to prevent the progressive decline of populations and mitigate current and future effects of climate change on *P*. *alabamensis* are considered and developed rapidly. There are currently no management and conservation initiatives being carried out throughout the species range or even for some populations, so the first step to ensure the survival of this species should be population and habitat monitoring. Head‐start programs for this species have been proposed (D. Nelson, personal communication), but never funded. Local monitoring activities to ensure habitat protection of the few sites where the species occurs, the potential development of a head‐start program for genetically pure *P*. *alabamensis* individuals, education of local citizens on the consequences of translocating and moving turtles to different water bodies (including species such as *Trachemys scripta* that can potentially compete with *P*. *alabamensis* for resources), maintenance of nesting sites, assessment of recruitment throughout the species’ range, and monitoring of population sizes should therefore be developed for this species.

## AUTHOR CONTRIBUTIONS

**Nickolas Moreno:** Data curation (lead); Formal analysis (lead); Resources (equal); Writing – original draft (equal); Writing – review & editing (equal). **Andrew Heaton:** Data curation (supporting); Resources (supporting); Writing – review & editing (supporting). **Kaylin Bruening:** Resources (supporting). **Emma Milligan:** Resources (supporting). **David Nelson:** Conceptualization (supporting); Resources (supporting); Writing – review & editing (supporting). **Scott Glaberman:** Conceptualization (lead); Formal analysis (supporting); Investigation (equal); Methodology (equal); Resources (supporting); Supervision (equal); Writing – review & editing (equal). **Ylenia Chiari:** Conceptualization (lead); Formal analysis (supporting); Funding acquisition (lead); Investigation (equal); Methodology (equal); Project administration (lead); Resources (lead); Supervision (equal); Writing – original draft (equal); Writing – review & editing (equal).

## CONFLICT OF INTEREST

None of the authors have competing interests.

## Data Availability

Haplotype sequence data have been deposited in the NCBI GenBank (accession numbers: MZ966274–MZ966297). Microsatellite genotyping for each individual and each locus are provided in Supplementary Data S1; *Q* assignment values are provided in Supplementary Data S2. Supplementary Data 1: Microsatellite genotyping for each individual and each locus. “‐9” refers to missing data. Supplementary Data 2: *Q* assignment values for each individual with respect to each different species and *P*. *alabamensis* clusters (Alabama vs. Mississippi). Each tab indicates the Q values for specific comparisons (e.g., *P*. *alabamensis* vs. *P*. *concinna*). Supplementary Data 1 and 2 can be found on Dryad at https://doi.org/10.5061/dryad.xpnvx0kj2.
