# Corrigendum

**DOI:** 10.1002/ece3.9059

**Published:** 2022-06-24

In the recent article by Moreno et al. (2022), the reference citation “Heaton et al. 2021” should read “Heaton et al. 2022,” and in the reference section, it should read “Heaton, A. J., Goff, J. A., Cressman, K. A., and Pitchford, J. L. (2022). The response of a pine savanna herpetofauna community to habitat health and restoration. *Herpetological Conservation and Biology*, **17**(1), 165–179.” The publication year was corrected from 2021 to 2022.

The authors apologize for the error.
